# Remote self-administration of digital cognitive tests using the Brief Assessment of Cognition: Feasibility, reliability, and sensitivity to subjective cognitive decline

**DOI:** 10.3389/fpsyt.2022.910896

**Published:** 2022-08-24

**Authors:** Alexandra S. Atkins, Michael S. Kraus, Matthew Welch, Zhenhua Yuan, Heather Stevens, Kathleen A. Welsh-Bohmer, Richard S. E. Keefe

**Affiliations:** ^1^WCG-VeraSci, Durham, NC, United States; ^2^Duke University Medical Center, Durham, NC, United States

**Keywords:** remote assessment, digital biomarkers, subjective cognitive decline, cognition, digital endpoints

## Abstract

Cognitive impairment is a common and pervasive feature of etiologically diverse disorders of the central nervous system, and a target indication for a growing number of symptomatic and disease modifying drugs. Remotely acquired digital endpoints have been recognized for their potential in providing frequent, real-time monitoring of cognition, but their ultimate value will be determined by the reliability and sensitivity of measurement in the populations of interest. To this end, we describe initial validation of remote self-administration of cognitive tests within a regulatorily compliant tablet-based platform. Participants were 61 older adults (age 55+), including 20 individuals with subjective cognitive decline (SCD). To allow comparison between remote (in-home) and site-based testing, participants completed 2 testing sessions 1 week apart. Results for three of four cognitive domains assessed demonstrated equivalence between remote and site-based tests, with high cross-modality ICCs (absolute agreement) for Symbol Coding (ICC = 0.75), Visuospatial Working Memory (ICC = 0.70) and Verbal Fluency (ICC > 0.73). Group differences in these domains were significant and reflected sensitivity to objective cognitive impairment in the SCD group for both remote and site-based testing (*p* < 0.05). In contrast, performance on tests of verbal episodic memory suggested inflated performance during unmonitored testing and indicate reliable use of remote cognitive assessments may depend on the construct, as well as the population being tested.

## Objective

Describe the feasibility, reliability, and sensitivity of remote self-administration of brief cognitive tests of processing speed, visuospatial working memory, verbal fluency and episodic verbal memory in older adults with and without subjective cognitive decline using an FDA/EMA compliant testing platform.

## Introduction

Cognitive impairment is a common, pervasive, and undertreated feature of neurologic and psychiatric disease that incurs significant costs in the form of lost wages, hospitalization and extended long-term care. In Alzheimer’s disease and related disorders (ADRD), self and/or partner-reported declines in the ability to remember, pay attention and/or engage in complex visuospatial tasks often represent the first overt sign of an advanced disease process that has silently progressed over a putatively asymptomatic preclinical period lasting years or decades prior to symptom onset (1, 2). Once manifest, continued declines in cognition, often occurring over the course of many years, predict the onset of functional impairments (3–5), ultimately leading to profound disability and loss of life.

This predictive relationship between cognitive decline and real-world functioning is not unique to Alzheimer’s disease (AD). Cognitive impairment is now recognized as a core or common feature and source of functional impairment in an etiologically diverse set central nervous system (CNS) disorders, including multiple sclerosis (MS) (6–8), Parkinson’s disease (9–12), schizophrenia (13–18), major depression (19) and bipolar disorder (20–22), among others. In MS, for instance, impairments in processing speed and executive functioning are common early symptoms associated with disease progression and relapse, the onset of which may precede clinical manifestation of motor symptoms (6–8). In Parkinson’s disease, subjective and subtle objective cognitive impairment has been shown to predict diagnosis by up to 9 years, and cognitive decline is now recognized within the emerging research framework for prodromal disease (9–12, 23). In symptomatic PD, as in other neurodegenerative diseases, cognitive impairment is associated with reductions in functional independence and with increases medical costs, caregiver burden and mortality (24).

Interactions between cognitive impairment and functional disability are similarly characterized in non-degenerative psychiatric disorders, most notably in schizophrenia (14, 17, 20, 25–28), bipolar disorder (20–22), and major depression (19). In each case, evidence suggests persistence of cognitive deficits impacting function despite effective mediation and/or resolutions of primary mood symptoms.

Given the real-world economic and societal impact of functional disability associated with cognitive impairment in CNS disorders, the value of cognitive impairment as a treatment target in drug development is clear. With respect to measurement of early or subtle cognitive declines, however, clinical outcome assessments (COAs) comprising traditional efficacy endpoints may lack sensitivity to change, particularly in preclinical or early prodromal disease (29), and may fail to adequately capture the real-world impact of emerging cognitive declines. Development of digital measures with increased precision have been proposed to fill this gap, improving sensitivity to intraindividual change through long-term monitoring of those at risk (30–33). This may be best achieved by leveraging the combined potential of *passive sensor-based* measurements of constructs such as gait, sleep and walking speed (31) and *active performance-based* ecological momentary assessments (EMAs), including brief repeatable cognitive tests (30, 34, 35). These *active digital markers* serve a dual purpose, offering direct, real-time performance-based assessment of cognitive domains of interest while providing contextual benchmarks to assist with the development, interpretation, and eventual validation of passively derived digital signatures.

Within this context, the present study sought to evaluate initial feasibility, reliability, and sensitivity of brief, self-administered cognitive tests adapted from clinically validated measures within the Brief Assessment of Cognition (BAC) and remotely delivered using existing DHT software (Pathway ePRO). As a rater administered measure, assessments within the BAC battery have demonstrated equivalency to original pen-and-paper measures (36) and shown sensitivity to cognitive impairment in schizophrenia, subjective cognitive decline (SCD) and MCI (36–38). The BAC includes voice-over instructions, automated stimulus presentation, integrated scoring, automatic data upload and cloud-based data storage in compliance with FDA 21CRF Part 11. Self-administered versions of BAC processing speed (Symbol Coding), visuospatial working memory, verbal fluency, and episodic verbal memory were developed to allow remote self-administration.

## Materials and methods

### Participants

Participants were 61 older adults aged 55 and above, including 41 healthy controls (HC) and 20 participants who endorsed moderate levels of SCD based on a total score ≥ 4 on the self-reported Cognitive Functional Instrument (CFI); (39). Participants were primarily recruited using an existing database maintained by WCG-VeraSci that includes individuals who have either participated in past research and/or expressed interest in future participation. Additional participants were recruited using IRB-approved digital advertisements and printed flyers.

All participants were fluent in English, non-demented and absent known neurological or psychiatric disease. Additional inclusion requirements for participants in the HC group included MMSE ≥ 24 for participants with ≥ 13 years of formal education, or MMSE ≥ 22 for those with < 13 years of education. For participants in the SCD group, requirements were MMSE ≥ 22 for participants with ≥ 13 years of education and MMSE ≥ 20 for others. Additional exclusionary criteria included the presence of sensory or motor deficits that would interfere with cognitive testing, current or recent diagnosis of alcohol or substance abuse, and/or daily use of illicit drugs or cannabinoids. Participants were compensated at a rate of $50/visit, for both in-person and remote visits. Participants who completed all visits received total compensation of $150.

The study protocol and informed consent were approved by the WIRB-Copernicus Group. All participants provided written informed consent prior to completing any study-related activities.

Participant demographics and baseline characteristics are displayed in Table 1.

**TABLE 1:** Participant characteristics.

	HC *n* = 41		SCD *n* = 20			
	Mean	SD	Mean	SD	*t*	*P*-value
Age (years)	67.02	7.71	70.30	9.76	–1.43	ns
Education (years)	16.02	2.60	16.05	2.30	–0.04	ns
MMSE	28.17	1.59	27.20	1.37	2.35	<0.05
CFI*	1.51	1.11	6.48	2.51	–8.44	<0.001
ADCS-ADL-PI*	42.88	2.51	38.05	5.07	4.04	<0.001

	***n* (%)**		***n* (%)**		**χ^2^**	***p***

Sex					0.44	ns
Male	18 (43.9%)		7 (35.0%)			
Female	23 (56.1%)		13 (65.0%)			
Race					0.90	ns
White	31 (75.6%)		14 (70%)			
African American	9 (22.0%)		6 (30%)			
Other	1 (2.4%)		0 (0.0%)			

*Self-reported measure.

### Measures

#### Self-administered Brief Assessment of Cognition

Detailed descriptions of the rater-administered BAC have been provided elsewhere (36). In selecting and revising standard BAC assessments for remote self-administration, our goal was to maximize sensitivity to early cognitive declines in ADRD while minimizing the time and burden to participants. Descriptions of each self-administered BAC cognitive test are provided in Table 2, and reflect the following adaptations to the standard rater-administered measures:

**TABLE 2:** Brief Assessment of Cognition (BAC) self-administered digital cognitive tests.

Domain	Test name	Description
Episodic verbal memory	3-trial verbal memory (Learning)	Subject hears 15 unrelated words and is asked to recall as many as possible. This procedure is repeated 3 times. *Duration*: 7 min *Outcome measure*: Total number of words recalled (Trials 1–3) *Alternate forms*: 7
	Delayed Recall	Following a standard delay, subject is asked to recall as many words as possible from the previous list. *Duration:* 2 min *Outcome measure:* Number of words recalled *Alternate forms*: 7
Working memory	Visuospatial working memory–sequences	Subject is presented with progressively longer series of objects placed within a grid. Memory for the location of each object is queried in sequence. *Duration*: 4–5 min *Outcome measure:* Number of correct sequences (all locations recalled) *Alternate forms*: 2
Verbal fluency	Animal fluency	Subject is given 60 s to name as many animals as possible. *Duration*: 2 min *Outcome measure:* Total numbers of correct words generated
Speed of processing	Symbol Coding	Subject is provided a key and asked to fill in the corresponding numbers beneath a series of symbols as quickly as possible within 90 s *Duration*: 3 min *Outcome measure:* Number of correct items *Alternate forms*: 8

Self-administered BAC tests represent modified, abbreviated versions of standard rater-administered assessments. Each test can be completed individually or as a part of a battery that includes additional performance-based assessments, PROs or ecological momentary assessments.

##### Verbal memory

The standard BAC measure includes an assessment of verbal memory that is designed to maximize sensitivity to deficits in verbal learning associated with schizophrenia. As such, the measure includes a relatively large number repeated learning trials (5 total) in which participants hear and attempt to recall 15 unrelated words (40, 41). More recently a Delayed Recall trial has been incorporated to increase sensitivity to deficits in delayed episodic retrieval from verbal memory (42). In order to balance the need for meaningful assessment of both verbal learning and delayed recall in SCD/ADRD with the risk of participant burden and disengagement associated with extended task length, the self-administered version of BAC Verbal Memory was modified to include a total of 3 learning trials completed at the beginning of the battery, and a single delayed recall trial completed at the end.

##### Verbal fluency

Traditional administration of the standard BAC measure includes 2 trials of letter fluency (F,S) and one trial of semantic fluency (animal naming); these are summed to produce a single measure of combined phonemic and semantic (category) fluency. In MCI and Mild AD, a preponderance of evidence suggests a strong advantage for semantic fluency in detection of early cognitive declines associated with AD pathology (43–46). Considering this, only semantic fluency was included in the self-administered version of BAC fluency.

##### Visuospatial WM

This assessment constitutes an addition to the standard BAC, introduced as a digital measure to increase sensitivity to impairments in visuospatial processing and spatial working memory. In its original form, there are two phases to this task. During Phase 1, locations of visuospatial memoranda are probed sequentially in the order of appearance (see Table 2). In Phase 2, sequences are probed in random order, requiring subjects to recall the precise item-location pair. More recently, Phase 1 has been utilized independently and demonstrated similar sensitivity. In accordance with this work, only Phase 1 is included in the self-administered task.

##### Symbol Coding

No substantive changes.

### Tablet orientation and practice

At the first self-administered testing session (either on-site or remote), participants were presented with a 4 to 5-min interactive orientation module covering basic use of the tablet-based technology, including basic instructions and practice tapping, swiping, entering/recording responses and following voice-over instructions. At the introduction to each cognitive test, participants heard spoken test instructions and completed practice items to ensure understanding prior to formal testing. During practice, corrective feedback was provided as needed, both visually and through voiced over corrective messaging. Participants were given the option to repeat instructions and practice as needed or desired prior to moving on to the test.

#### Mini mental state exam

The mini mental state exam (MMSE; 50) is a brief, standardized cognitive screening instrument used widely in research and clinical practice. The MMSE was administered by a trained rater at the screening visit.

#### Cognitive function instrument

The cognitive function instrument (CFI; 39, 51) is a brief, 14-item questionnaire that can be completed by participants or a caregiver to assess SCD over the past year. The self-completed version is used in this study. Total CFI scores range 0–14, with higher scores indicating higher levels of subjective decline. In prior studies, individuals endorsing similar levels of SCD (CFI total ≥ 4) have demonstrated increased risk for beta amyloid positivity, increased risk for progression to symptomatic disease, and impaired performance on cognitive testing using the rater-administered BAC (51–55, 37).

#### Alzheimer’s Disease Cooperative Study – Activities of Daily Living, Prevention Instrument

The Alzheimer’s Disease Cooperative Study – Activities of Daily Living, Prevention Instrument (ADCS-ADL-PI; 56) is a 20-item questionnaire that allows for self- and/or partner-reported assessment of basic and instrumental activities of daily living (ADLs and iADLs, respectively).

#### Participant Feedback Questionnaire

Following first self-administration of the BAC, participants were asked to provide subjective feedback on their experience using the tablet. Participants tapped response buttons labeled “strongly disagree,” “disagree,” “neither agree nor disagree,” “Agree,” or “strongly agree” in response to statements regarding their ability to (1) see text and objects clearly on the screen, (2) hear audio instructions clearly and (3) easily understand the audio instructions given throughout testing. In addition, participants were asked to rate their overall experience using the table on a scale of 1 (extremely difficult) to 10 (extremely easy).

##### Procedure

Rater-administered and participant-completed measures were collected using the Pathway ePRO/eCOA platform, an FDA-Part 11 compliant system that supports secure collection of rater-administered scales, patient-completed cognitive tests, ecological momentary assessments (EMAs) and participant/informant-based questionnaires. All study coordinators and raters were employees of WCG-VeraSci who were trained in Good Clinical Practices and certified in the administration and scoring of cognitive measures.

All participants attended 2 on-site study visits at the WCG-VeraSci Innovation Lab in Durham, NC. During Visit 1, participants completed informed consent, MMSE and eligibility screening. Following screening, eligible participants received brief, in-person training on the use of study devices (iPads) to be used for self-administration of cognitive tests and questionnaires using the Pathway App. During device credentialing, participants chose a unique PIN, which provided access to the Pathway App. If forgotten, this PIN could be reset remotely by the participant used standard dual-authentication procedures. During on-site Visits 1 and 2, participants completed an additional digital performance-based measure of functional capacity (the VRFCAT) and were trained in the use of an actigraphy monitor; these findings will be discussed elsewhere.

Participants were pseudo-randomly assigned, in equal numbers, to the order of remote vs. on-site cognitive testing sessions. Sessions were completed approximately 4–7 days apart, with 50% of participants completing on-site testing first (Sequence A) and 50% completing remote testing first (Sequence B). Those assigned to “Sequence A” completed on-site cognitive testing at on-site Visit 1 and remote testing 4–5 days later. Those assigned to “Sequence B” completed their first test session remotely 4–5 days following Visit 1 and on-site testing at on-site Visit 2 the following week. Alternate forms of Verbal Memory, Symbol Coding and Visuo spatial working memory were completed at each session. Following their first cognitive testing session (remote or in-person), participants completed the Participant Feedback Questionnaire and were asked to provide information regarding personal use of digital technologies including tablets and smartphones.

#### Statistical analysis

Statistical analyses were completed in R© and IBM SPSS 27©. Group differences in age, education, MMSE and self-reported scales were evaluated using two-tailed, between subjects *t*-tests.

Psychometric reliability of cognitive test performance across remote and site-based sessions was assessed using intraclass correlation coefficients (ICCs). ICCs were calculated using a two-way random-effects model (ICC2).

Site-based vs. remote test comparisons of raw scores were evaluated for the combined HC and SCD sample using a linear mixed effects model controlling for age and education, with testing order as a between subjects’ variable. Effect sizes for linear mixed effects are reported as standardized beta weights. *Post hoc* tests examined performance differences between HC and SCD groups, using Cohen’s *d* estimates of effect size.

Correlations between subjective measures of cognition (CFI) and ADL function (ADCS-ADL-PI) and between CFI, MMSE and BAC cognitive measures were calculated using Pearson’s product-moment-correlations.

## Results

Participant demographics and mean MMSE, CFI and ADCS-ADL-PI scores are provided in Table 1. Participants were predominantly female, with women comprising 56% of the HC group and 65% of the SCD group. The sample reflected moderate racial diversity, with approximately 30% of participants identifying as African American. Although the SCD group was slightly older on average, this difference did not reach significance (*p* > 0.1).

Group differences in MMSE total scores were statistically significant at Visit 1, indicating worse objective cognitive performance by those with SCD. As expected, based on inclusion criteria, CFI scores were significantly higher for those in the SCD group. Self-reported ADLs also differed by group, with those in the SCD group reporting greater impairment (Table 1). CFI and ADCS-ADL measures were significantly correlated across the sample (*r* = 0.70, *p* < 0.001) suggesting consistency across these two self-report measures.

Daily use of tablet or smart-phone devices was reported by 86.2% of participants, including 87.8% of HC and 82.4% of participants with SCD. Three participants, including two HCs, reported device usage less than one time per month.

ICCs reflecting absolute agreement between on-site and remote testing were calculated using raw data for the pooled sample, and for the HC and SCD groups individually (Table 3). ICCs for Symbol Coding, Verbal Fluency were strong (>0.7 for all) reflecting the test-retest reliability of the original BAC measures (36, 40). ICCs for measures of verbal memory were lower, and potentially impacted by the a small number of outliers reflecting substantially higher on remote versus in-person testing. On Verbal Memory Total Learning, a single outlier in the HC group performed 23 points higher during remote versus on-site testing. On Delayed Free recall, this individual and two additional HC outliers were identified, as well as a single extreme outlier in the SCD group. As with Total Learning, outliers on Delayed Recall reflected substantially higher performance during remote compared to on-site testing.

**TABLE 3:** ICC absolute agreement between remote and site-based measures.

Test	ICC (95% confidence interval)		
	Total sample	HC	SCD
Symbol Coding	0.747 (0.610, 0.841)	0.714 (0.521, 0.838)	0.78 (0.522, 0.907)
Visuospatial WM	0.733 (0.583, 0.833)	0.673 (0.459, 0.814)	0.786 (0.542, 0.909)
Verbal fluency	0.748 (0.610, 0.842)	0.75 (0.574, 0.86)	0.733 (0.436, 0.885)
Verbal memory–Total learning	0.478 (0.248, 0.658)	0.408 (0.11, 0.643)	0.548 (0.115, 0.804)
Verbal memory–Total learning (trimmed)*	0.579 (0.371, 0.733)	0.56 (0.29, 0.75)	n/a
Delayed Free Recall	0.247 (–0.01, 0.477)	0.264 (–0.055, 0.542)	0.154 (–0.309, 0.559)
Delayed Free Recall (trimmed)*	0.49 (0.246, 0.676)	0.544 (0.241, 0.751)	0.385 (–0.102, 0.718)

ICCs reflect use of alternate forms for Symbol Coding, Visuospatial WM and Verbal memory.

*“Trimmed” values reflect ICCs following removal of extreme outliers.

To evaluate the potential impact of outliers on ICCs for BAC assessments of verbal memory, ICCs were recalculated with outliers censored. ICCs based on this “trimmed” data are presented in Table 3 for comparison.

**FIGURE 1:**
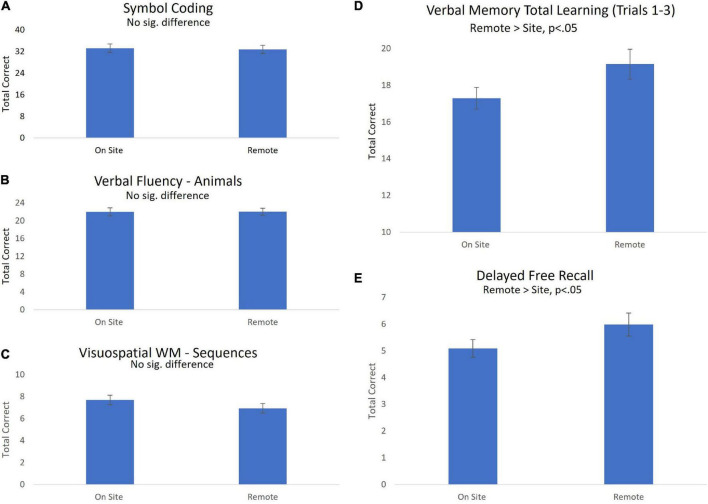
Performance on site-based vs. remote BAC cognitive tests (Mean ± SEM). Performance on Symbol Coding. **(A)** Verbal fluency **(B)** and Visuospatial WM **(C)** was similar for site-based and remote testing. Performance on measures of episodic verbal memory **(D,E)** were higher during remote testing, suggesting inflated performance during remote, unmonitored testing.

Figure 1 displays mean (± SEM) performance for each self-administered cognitive test during on-site and remote testing. Covariates for testing order did not approach significance for any test. No performance differences between site and remote assessments were observed for Symbol Coding, Verbal Fluency or Visuospatial WM (Figure 1, panels A–C) suggesting no significant variability in performance associated with remote vs. on site testing. In contrast, performance on Verbal Memory Total Learning and Delayed Free Recall were higher for remote compared to onsite testing (*b* = 1.88, *t* = 2.60, *p* < 0.05 for Total learning; *b* = 1.04, *t* = 2.23, *p* < 0.05 Delayed Free Recall; Figure 1, panels D,E).

**FIGURE 2:**
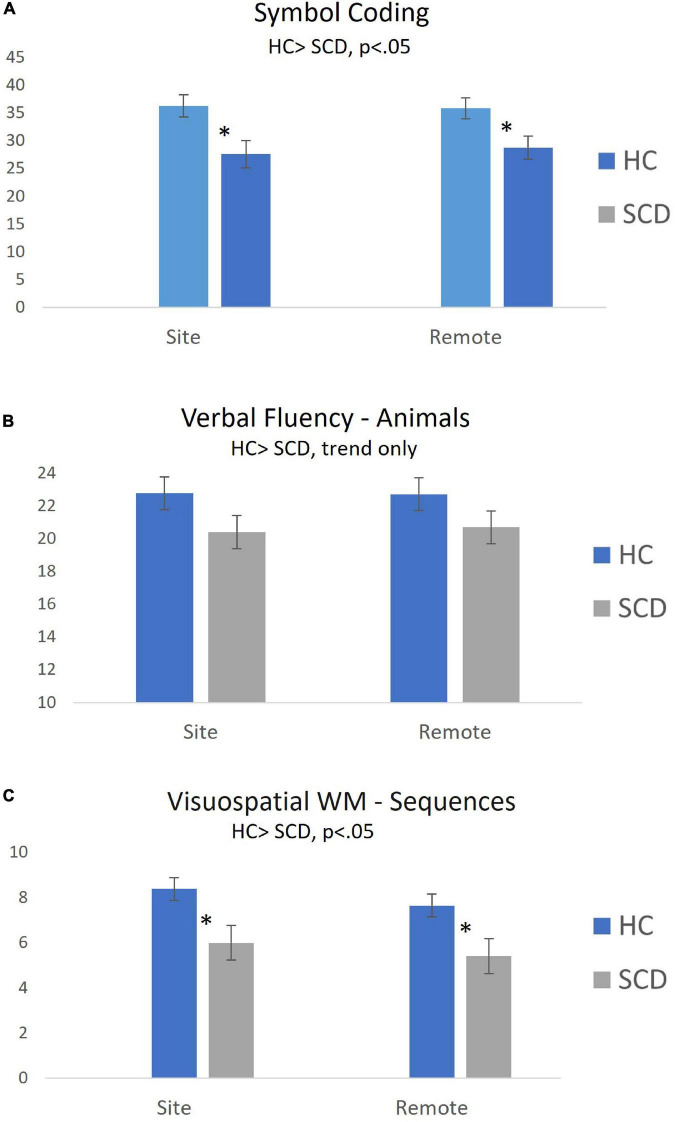
Group differences in cognitive test performance during on-site and remote testing sessions (Mean ± SEM). Self-administered remote and site-based assessments of processing speed [Symbol Coding; **(A)**] and Visuospatial WM **(C)** were equally sensitive to objective cognitive declines in participants with SCD. Group differences in Verbal Fluency **(B)** were similar for site-based and remote tasks, but did not reach statistical significance. *p < 0.05 for between-group comparison.

Figure 2 displays mean (± SEM) performance by Group (HC vs. SCD) for Symbol Coding, Verbal Fluency and Visuospatial WM. Performance differences between participants in the HC and SCD groups during site-based and remote testing were examined using *post hoc* pairwise comparisons. Significant Group differences were observed on Symbol Coding (*t* = 2.31, *p* < 0.05, *d* = 0.64 for Site; *t* = 2.04, *p* < 0.05, *d* = 0.56 for Remote), Visuospatial WM (*t* = 2.23, *p* < 0.05, *d* = 0.61 for Site; *t* = 2.16, *p* < 0.05, *d* = 0.59 for Remote), and Verbal Memory Total Learning (*t* = 2.38, *p* < 0.05 for Site, *d* = 0.68; *t* = 2.40, *p* < 0.05, *d* = 0.68 for Remote).

Exploratory analyses examined associations between CFI, MMSE and BAC cognitive measures were using Pearson’s correlations. Significant correlations were observed between the CFI and MMSE (*r* = 3.30, *p* < 0.001) and between the MMSE and several BAC measures (see Supplementary Table 1). For most measures, correlations were of similar magnitude for remote and on-site testing. A notable exception to this was the correlations between MMSE and Delayed Recall (*r* = 3.16, *p* < 0.05 for on-site BAC; *r* = 0.144, ns for remote BAC).

Responses to the Participant Feedback Questionnaire are provided in Table 4 which provides means and SD for rating provided by participants in the HC and SCD groups. Across the combined sample, 98.3% of participants provided ratings of “Strongly Agree” or “Agree” to items 1–3 regarding their ability to accurately see, hear and understand tablet-based screens and instructions. Mean ratings for both groups correspond with a response midway between “Strongly Agree” and “Agree”; no differences between groups were observed. Ratings of overall experience/ease of use were > 8 for both groups (10-point scale) but were significantly higher for participants in the HC group (*p* < 0.05).

**TABLE 4:** Participant feedback on Brief Assessment of Cognition (BAC) self-administration.

	HC		SCD			

Item*	Mean	SD	Mean	SD	*t*	*P*-value
1. See text and objects clearly	4.585	0.499	4.529	0.514	0.385	ns
2. Hear instructions clearly	4.585	0.547	4.588	0.507	–0.019	ns
3. Understand instructions easily	4.537	0.505	4.529	0.514	0.049	ns
4. Overall Experience (1–10)	9.195	1.054	8.412	1.583	2.210	*p* < 0.05

*Responses to items 1–3 are coded 1–5 (strongly disagree – strongly agree). Responses to item 4 reflect participant ratings on a scale of 1–10 (extremely difficult–extremely easy).

## Discussion

Contemporaneous advances in biomedical research and mobile digital technologies have welcomed parallel advances in traditional biomarkers (fluid/imaging/genetic) on the one hand, and novel digital tools on the other. In the area of ADRD, (relative) scientific consensus regarding the relevant biological constructs (e.g., amyloid beta, *p*-tau), has facilitated relatively rapid integration of novel biomarkers into existing clinical development and regulatory frameworks. For digital technologies, recent Food and Drug Administration draft guidance to industry regarding Digital Health Technologies for Remote Data Acquisition in Clinical Investigations (47) provides a tentative roadmap for integration of these tools, including recommended verification, validation and usability testing for the intended context of use. The current study represents an example of preliminary validation of abbreviated, self-administered, remotely acquired adaptations of previously validated measures collected using established DHT software (Pathway eCOA/ePRO).

Within this framework, we examined psychometric reliability, sensitivity and ease of use associated with self-administered cognitive tests within the BAC platform (36). Older adults with and without SCD completed on-site and at-home remote self-administered testing, provided user feedback, and completed evaluations using standard measures and questionnaires including the MMSE, CFI and ADCS-ADL. Main findings and implications are discussed below.

### Reliability of remote vs. on-site self-administration of cognitive tests

Findings regarding the reliability of remote, at-home (unmonitored) self-administration of cognitive tests suggest the reliability of remotely acquired measures may depend on the cognitive domain being evaluated. ICCs for absolute agreement between on-site and remote self-administration of Symbol Coding, Verbal Fluency and Visuospatial WM were very strong (ICC > 0.7 for all in pooled sample, Table 3). Direct comparisons between on-site and remotely acquired scores identified no statistical difference between modalities. Further, examination of mean performance across contexts provides a strong demonstration of equivalence between remote and on-site measures within pooled sample (Figure 1) and within each group individually (Figure 2). Finally, observed alignment in sensitivity to reduced performance associated with SCD (discussed below), provides additional compelling evidence in support of the reliability of both on-site and remote use of self-administered versions of Symbol Coding, Verbal Fluency and Visuospatial WM.

In contrast, assessments of verbal memory, including both Verbal Memory Total Learning and Delayed Recall, were associated with relatively low ICCs (Table 3), suggesting that remote, *unmonitored* self-assessment may be less reliable in this cognitive domain. Although ICCs numerically improved following removal of extreme outliers (see “trimmed” measures, Table 3), confidence intervals associated with these estimates remained quite wide. Comparisons of within-subject performance on remote versus in-person assessments revealed significant performance improvements associated with remote testing (Figures 1D,E). Although it can be difficult to identify a single cause for inflated memory performance during remote testing, the most parsimonious explanation may be a tendency for individuals to “cheat” during unmonitored assessments, a phenomenon that has become more widely observed and acknowledged with the increased usage of telehealth-based cognitive screening during the era of COVID-19 (48). The phenomenon appears to be specifically problematic on verbal memory measures, measure that are typically among the most sensitive to early staged memory disorders (49). How best to detect and prevent cheating in remote assessment is a critical topic for future work in the field.

### Sensitivity to cognitive impairment and associations between measures

Participants in the SCD group were characterized by mild objective decline on the MMSE relative to the HC group (Table 1). On the BAC, participants with SCD performed significantly worse on in-person and remote measures of Symbol Coding, Visuospatial WM and Verbal Memory Total Learning (see results). Consistency of observed results suggests remote and on-site assessments were similarly sensitive to impairments in this sample.

Correlations between CFI and self-reported iADL/ADLs provide a consistent picture of the SCD group as exhibiting reduced levels of self-reported cognition and function. Significant correlations were also observed between the CFI and MMSE, and between the MMSE and several BAC measures (Supplementary Table 1). Taken together, these findings support characterization of participants in the SCD group as exhibiting subtle, sub-clinical deficits compared the HCs, and may provide support for SCD as an *ad hoc proxy* for AD risk in the absence of confirmative biomarkers.

### Participant feedback regarding ease of use

Eliciting feedback from populations of interest is critical to the development of digital tools, particularly for use cases that include direct use and management of digital technologies. In the current study, participant feedback was overwhelmingly positive regarding ease of use, including the ability to see, hear and understand visual and auditory information and instructions provided by the tablet (Table 4). It is important to interpret this feedback within the limitations of the current study, which included in-person support for the credentialing and initial training on the tablet-based platform. Although encouraging, it will be important to determine the level of support required for implementing broader use cases that may include remote deployment of technologies or BYOD methods.

### Limitations and future implications

Some limitations of the current work include the relatively small sample size, particularly in the SCD group, the high education level of the sample, and the restricted MMSE range of those included. Although there was moderate racial diversity to in the sample, including 30% participation by individuals self-identified as black or African American, there was limited representation from other racial and sociocultural groups. In order to best understand the generalizability of findings as they relate to remote self-administration of cognitive assessment, deployment of measures to a wider, more diverse sample is required.

Despite limitations, the current findings are encouraging in demonstrating the feasibility, reliability, and sensitivity of remote, home-based self-assessment of cognition by older adults across several cognitive domains. More broadly, results highlight the strong potential of self-administered digital cognitive tests to improve detection and ongoing monitoring of cognitive decline in older adults within the context of clinical trials and clinical practice alike. Finally, results suggest that brief digital tests provide reliable and sensitive active digital markers that, when combined with data from passive sensor-based tools, may help inform our broader understanding of cognition and function in real-world settings.

## Data availability statement

The raw data supporting the conclusions of this article will be made available by the authors upon reasonable request, without undue reservation.

## Ethics statement

The studies involving human participants were reviewed and approved by WIRB-Copernicus Group. The patients/participants provided their written informed consent to participate in this study.

## Author contributions

AA led protocol development, wrote the manuscript, completed analyses, and interpreted findings. MK managed research operations, including participant recruitment, and screening and data collection. HS provided data management support. MW provided statistical analysis. ZY provided statistical analysis. KW-B provided subject matter input to protocol and manuscript. RK provided subject matter input to protocol and manuscript. All authors contributed to the article and approved the submitted version.

## References

[1] Jack CR Bennett DA Blennow K Carrillo MC Dunn B Haeberlein SB Nia-Aa research framework: toward a biological definition of Alzheimer’s disease. *Alzheimers Dement.* (2018) 14:535–62. 10.1016/j.jalz.2018.02.018 29653606 PMC5958625

[2] Hardy JA Higgins GA. Alzheimer’s disease: the amyloid cascade hypothesis. *Science.* (1992) 256:184–5. 10.1126/science.1566067 1566067

[3] Liu-Seifert H Siemers E Price K Han B Selzler KJ Henley D Cognitive impairment precedes and predicts functional impairment in mild Alzheimer’s disease. *J Alzheimers Dis.* (2015) 47:205–14. 10.3233/jad-142508 26402769 PMC4923754

[4] Liu-Seifert H Siemers E Sundell K Price K Han B Selzler K Cognitive and functional decline and their relationship in patients with mild Alzheimer’s dementia. *J Alzheimers Dis.* (2015) 43:949–55. 10.3233/jad-140792 25125457

[5] Belleville S Fouquet C Hudon C Zomahoun HTV Croteau J. Consortium for the early identification of Alzheimer’s d-q. neuropsychological measures that predict progression from mild cognitive impairment to Alzheimer’ type dementia in older adults: a systematic review and meta-analysis. *Neuropsychol Rev.* (2017) 27:328–53. 10.1007/s11065-017-9361-5 29019061 PMC5754432

[6] Baughman BC Basso MR Sinclair RR Combs DR Roper BL. Staying on the job: the relationship between work performance and cognition in individuals diagnosed with multiple sclerosis. *J Clin Exp Neuropsychol.* (2015) 37:630–40. 10.1080/13803395.2015.1039963 26149071 PMC11391886

[7] Benedict RHB Amato MP DeLuca J Geurts JJG. Cognitive impairment in multiple sclerosis: clinical management, MRI, and therapeutic avenues. *Lancet Neurol.* (2020) 19:860–71. 10.1016/S1474-4422(20)30277-5 32949546 PMC10011205

[8] Cortese M Riise T Bjørnevik K Bhan A Farbu E Grytten N preclinical disease activity in multiple sclerosis: a prospective study of cognitive performance prior to first symptom. *Ann Neurol.* (2016) 80:616–24. 10.1002/ana.24769 27554176

[9] Darweesh SKL Wolters FJ Postuma RB Stricker BH Hofman A Koudstaal PJ Association between poor cognitive functioning and risk of incident Parkinsonism. *JAMA Neurol.* (2017) 74:1431. 10.1001/jamaneurol.2017.2248 28973176 PMC5822187

[10] Heinzel S Berg D Gasser T Chen H Yao C Postuma RB. Update of the MDS research criteria for prodromal Parkinson’s Disease. *Mov Disord.* (2019) 34:1464–70. 10.1002/mds.27802 31412427

[11] Hinkle JT Perepezko K Bakker CC Dawson TM Johnson V Mari Z Domain-specific cognitive impairment in non-demented Parkinson’s disease psychosis. *Int J Geriatr Psychiatry.* (2018) 33:e131–9. 10.1002/gps.4736 28509347 PMC5698175

[12] Speelberg DHB Janssen Daalen JM Bloem BR Gagnon J-F Post B Darweesh SKL. Prodromal cognitive deficits and the risk of subsequent Parkinson’s disease. *Brain Sci.* (2022) 12:199. 10.3390/brainsci12020199 35203962 PMC8870093

[13] Green MF Horan WP Lee J. Nonsocial and social cognition in schizophrenia: current evidence and future directions. *World Psychiatry.* (2019) 18:146–61. 10.1002/wps.20624 31059632 PMC6502429

[14] Green MF Kern RS Braff DL Mintz J. Neurocognitive deficits and functional outcome in schizophrenia: are we measuring the “right stuff”? *Schizophr Bull.* (2000) 26:119–36. 10.1093/oxfordjournals.schbul.a033430 10755673

[15] Harvey PD Horan WP Atkins AS Stevens H Welch M Yuan J Factor structure of cognitive performance and functional capacity in schizophrenia: evidence for differences across functional capacity measures. *Schizophr Res.* (2020) 223:297–304. 10.1016/j.schres.2020.08.010 32928621 PMC7704623

[16] Kadakia A Fan Q Shepherd J Dembek C Bailey H Walker C Healthcare resource utilization and quality of life by cognitive impairment in patients with schizophrenia. *Schizophr Res Cogn.* (2022) 28:100233. 10.1016/j.scog.2021.100233 35004189 PMC8715204

[17] Keefe RS Poe M Walker TM Harvey PD. The relationship of the brief assessment of cognition in schizophrenia (bacs) to functional capacity and real-world functional outcome. *J Clin Exp Neuropsychol.* (2006) 28:260–9. 10.1080/13803390500360539 16484097

[18] Sevy S Davidson M. The cost of cognitive impairment in schizophrenia. *Schizophr Res.* (1995) 17:1–3. 10.1016/0920-9964(95)00025-h 8541242

[19] Jaeger J Berns S Uzelac S Davis-Conway S. Neurocognitive deficits and disability in major depressive disorder. *Psychiatry Res.* (2006) 145:39–48. 10.1016/j.psychres.2005.11.011 17045658

[20] Bowie CR Best MW Depp C Mausbach BT Patterson TL Pulver AE Cognitive and functional deficits in bipolar disorder and schizophrenia as a function of the presence and history of psychosis. *Bipolar Disord.* (2018) 20:604–13. 10.1111/bdi.12654 29777563

[21] Jaeger J Berns S Loftus S Gonzalez C Czobor P. Neurocognitive test performance predicts functional recovery from acute exacerbation leading to hospitalization in bipolar disorder. *Bipolar Disord.* (2007) 9:93–102. 10.1111/j.1399-5618.2007.00427.x 17391353

[22] Wingo AP Harvey PD Baldessarini RJ. Neurocognitive Impairment in bipolar disorder patients: functional implications. *Bipolar Disord.* (2009) 11:113–25. 10.1111/j.1399-5618.2009.00665.x 19267694

[23] Fereshtehnejad S-M Yao C Pelletier A Montplaisir JY Gagnon J-F Postuma RB. Evolution of prodromal Parkinson’s disease and dementia with lewy bodies: a prospective study. *Brain.* (2019) 142:2051–67. 10.1093/brain/awz111 31111143

[24] Gonzalez-Latapi P Bayram E Litvan I Marras C. Cognitive impairment in Parkinson’s disease: epidemiology, clinical profile, protective and risk factors. *Behav Sci.* (2021) 11:74. 10.3390/bs11050074 34068064 PMC8152515

[25] Lewandowski KE Cohen BM Keshavan MS Sperry SH Ongur D. Neuropsychological functioning predicts community outcomes in affective and non-affective psychoses: a 6-month follow-up. *Schizophr Res.* (2013) 148:34–7. 10.1016/j.schres.2013.05.012 23791391 PMC3751391

[26] Brosey E Woodward ND. Schizotypy and clinical symptoms, cognitive function, and quality of life in individuals with a psychotic disorder. *Schizophr Res.* (2015) 166:92–7. 10.1016/j.schres.2015.04.038 26002072

[27] Fu S Czajkowski N Rund BR Torgalsbøen AK. The relationship between level of cognitive impairments and functional outcome trajectories in first-episode schizophrenia. *Schizophr Res.* (2017) 190:144–9. 10.1016/j.schres.2017.03.002 28302394

[28] Sheffield JM Kandala S Tamminga CA Pearlson GD Keshavan MS Sweeney JA Transdiagnostic associations between functional brain network integrity and cognition. *JAMA Psychiatry.* (2017) 74:605–13. 10.1001/jamapsychiatry.2017.0669 28467520 PMC5539843

[29] Schneider LS Goldberg TE. Composite cognitive and functional measures for early stage Alzheimer’s disease trials. *Alzheimers Dement (Amst).* (2020) 12:e12017. 10.1002/dad2.12017 32432155 PMC7233425

[30] Jutten RJ Rentz DM Fu JF Mayblyum DV Amariglio RE Buckley RF Monthly at-home computerized cognitive testing to detect diminished practice effects in preclinical Alzheimer’s disease. *Front Aging Neurosci.* (2021) 13:800126. 10.3389/fnagi.2021.800126 35095476 PMC8792465

[31] Dagum P. Digital biomarkers of cognitive function. *NPJ Digital Med.* (2018) 1:10. 10.1038/s41746-018-0018-4 31304295 PMC6550173

[32] Gold M Amatniek J Carrillo MC Cedarbaum JM Hendrix JA Miller BB Digital technologies as biomarkers, clinical outcomes assessment, and recruitment tools in Alzheimer’s disease clinical trials. *Alzheimers Dement (N Y).* (2018) 4:234–42. 10.1016/j.trci.2018.04.003 29955666 PMC6021547

[33] Wu CY Beattie Z Mattek N Sharma N Kaye J Dodge HH. Reproducibility and replicability of high-frequency, in-home digital biomarkers in reducing sample sizes for clinical trials. *Alzheimers Dement (N Y).* (2021) 7:e12220. 10.1002/trc2.12220 35005204 PMC8719347

[34] Gomar JJ Bobes-Bascaran MT Conejero-Goldberg C Davies P Goldberg TE. Utility of combinations of biomarkers, cognitive markers, and risk factors to predict conversion from mild cognitive impairment to Alzheimer disease in patients in the Alzheimer’s disease neuroimaging initiative. *Arch Gen Psychiatry.* (2011) 68:961–9. 10.1001/archgenpsychiatry.2011.96 21893661

[35] Chen L Asgari M Gale R Wild K Dodge H Kaye J. Improving the assessment of mild cognitive impairment in advanced age with a novel multi-feature automated speech and language analysis of verbal fluency. *Front Psychol.* (2020) 11:535. 10.3389/fpsyg.2020.00535 32328008 PMC7160369

[36] Atkins AS Tseng T Vaughan A Twamley EW Harvey P Patterson T Validation of the tablet-administered brief assessment of cognition (Bac App). *Schizophr Res.* (2017) 181:100–6. 10.1016/j.schres.2016.10.010 27771201

[37] AtkinsASKhanAUlshenDVaughanABalentinDDickersonH Assessment of instrumental activities of daily living in older adults with subjective cognitive decline using the virtual reality functional capacity assessment tool (Vrfcat). *J Prev Alzheimers Dis.* (2018) 5:216–24. 10.14283/jpad.2018.28 30298179

[38] Atkins AS. *Digital Assessments for Clinical Staging and Monitoring of Cognitive Performance in Early Symptomatic Ad.* Washington, DC: Alzheimer’s Association Research Roundtable (2021).

[39] Walsh SP Raman R Jones KB Aisen PS. Adcs prevention instrument project: the mail-in cognitive function screening instrument (MCFSI). *Alzheimer Dis Assoc Disord.* (2006) 20:S170–8. 10.1097/01.wad.0000213879.55547.57 17135810

[40] Keefe RS Goldberg TE Harvey PD Gold JM Poe MP Coughenour L. The brief assessment of cognition in schizophrenia: reliability, sensitivity, and comparison with a standard neurocognitive battery. *Schizophr Res.* (2004) 68:283–97. 10.1016/j.schres.2003.09.011 15099610

[41] Keefe RS Harvey PD Goldberg TE Gold JM Walker TM Kennel C Norms and standardization of the brief assessment of cognition in schizophrenia (Bacs). *Schizophr Res.* (2008) 102:108–15. 10.1016/j.schres.2008.03.024 18495435

[42] Atkins AS Khan A Welsh-Bohmer KA Plassman BL Randolph C Harrison J Expanding the brief assessment of cognition (Bac-App) for assessment of cognition in aging: initial findings from an ongoing normative study. *Alzheimers Dement.* (2017) 13:1574–5.

[43] Butters N Granholm E Salmon DP Grant I Wolfe J. Episodic and semantic memory: a comparison of amnesic and demented patients. *J Clin Exp Neuropsychol.* (1987) 9:479–97. 10.1080/01688638708410764 2959682

[44] Laws KR Duncan A Gale TM. ‘Normal’ semantic-phonemic fluency discrepancy in Alzheimer’s disease? A meta-analytic study. *Cortex.* (2010) 46:595–601. 10.1016/j.cortex.2009.04.009 19560132

[45] Monsch AU Bondi MW Butters N Salmon DP Katzman R Thal LJ. Comparisons of verbal fluency tasks in the detection of dementia of the Alzheimer type. *Arch Neurol.* (1992) 49:1253–8. 10.1001/archneur.1992.00530360051017 1449404

[46] Papp KV Mormino EC Amariglio RE Munro C Dagley A Schultz AP Biomarker validation of a decline in semantic processing in preclinical Alzheimer’s disease. *Neuropsychology.* (2016) 30:624–30. 10.1037/neu0000246 26595826 PMC4877295

[47] Food and Drug Administration. *Draft Guidance on Remote Data Acquisition in Trials.* Silver Spring, MD: Food and Drug Administration (2021).

[48] Ramsdale E. Lessons Learned from Covid-19: Telehealth/Physician Perspective. Drug Research and Development for Adults across the Older Age Span; Virtual Workshopnational Academies of Science, Engineering, and Medicine. (2020). Available online at: https://www.nationalacademies.org/event/08-05-2020/drug-research-and-development-for-older-adult-populations-a-workshop (accessed June 15, 2022).

[49] Welsh KA Butters N Hughes JP Mohs RC Heyman A. Detection of abnormal memory decline in mild Alzheimer’s disease using cerad neuropsychological measures. *Arch Neurol.* (1991) 48:278–81. 10.1001/archneur.1991.00530150046016 2001185

[50] Folstein MF Folstein SE McHugh PR. “Mini-mental state”. A practical method for grading the cognitive state of patients for the clinician. *J Psychiatr Res.* (1975) 12:189–98. 10.1016/0022-3956(75)90026-6 1202204

[51] Amariglio RE Donohue MC Marshall GA Rentz DM Salmon DP Ferris SH Tracking early decline in cognitive function in older individuals at risk for Alzheimer disease dementia: the Alzheimer’s disease cooperative study cognitive function instrument. *JAMA Neurol.* (2015) 72:446–54. 10.1001/jamaneurol.2014.3375 25706191 PMC4397164

[52] Li A Yue L Xiao S Liu M. Cognitive function assessment and prediction for subjective cognitive decline and mild cognitive impairment. *Brain Imaging Behav.* (2021) 16:645–58. 10.1007/s11682-021-00545-1 34491529

[53] Li C Neugroschl J Luo X Zhu C Aisen P Ferris S The utility of the cognitive function instrument (Cfi) to detect cognitive decline in non-demented older adults. *J Alzheimers Dis.* (2017) 60:427–37. 10.3233/jad-161294 28854503 PMC6417419

[54] Amariglio RE Becker JA Carmasin J Wadsworth LP Lorius N Sullivan C Subjective cognitive complaints and amyloid burden in cognitively normal older individuals. *Neuropsychologia.* (2012) 50:2880–6. 10.1016/j.neuropsychologia.2012.08.011 22940426 PMC3473106

[55] Amariglio RE Buckley RF Mormino EC Marshall GA Johnson KA Rentz DM Amyloid-associated increases in longitudinal report of subjective cognitive complaints. *Alzheimers Dement (N Y).* (2018) 4:444–9. 10.1016/j.trci.2018.08.005 30258973 PMC6153378

[56] Galasko D Bennett DA Sano M Marson D Kaye J Edland SD Alzheimer’s Disease Cooperative Study. ADCS Prevention Instrument Project: assessment of instrumental activities of daily living for community-dwelling elderly individuals in dementia prevention clinical trials. *Alzheimer Dis Assoc Disord.* (2006) 20:S152–69. 10.1097/01.wad.0000213873.25053.2b 17135809

