# Supplementary material for: Remote self-administration of digital cognitive tests using the Brief Assessment of Cognition: Feasibility, reliability, and sensitivity to subjective cognitive decline
Source: Front Psychiatry. 2022 Aug 24;13:910896. doi: 10.3389/fpsyt.2022.910896 (PMC9448897; doi:10.3389/fpsyt.2022.910896)
Supplement: Supplementary file 1 [file Table_1.docx]

| Site  Remote | MMSE | CFI | Verbal Memory - Total Learning | Delayed Free Recall | Visuospatial WM | Symbol Coding | Verbal Fluency |
| --- | --- | --- | --- | --- | --- | --- | --- |
| MMSE |  | **-.330**** | **.364^**^** | **.316*** | .180 | **.269*** | .164 |
| CFI | **-.330**** |  | -.281^*^ | -0.171 | -0.172 | **-.269^*^** | -.172 |
| Verbal Memory- Total Learning | **.321*** | -0.204 |  | .683^**^ | .282^*^ | **.364^**^** | **.395**** |
| Delayed Free Recall | .144 | -0.025 | **.721^**^** |  | 0.122 | **.393^**^** | **.374**** |
| Visuospatial WM | .158 | -0.142 | .337^*^ | 0.105 |  | **.429^**^** | **.308*** |
| Symbol Coding | .202 | -0.244 | **.444^**^** | **.368^**^** | **.450^**^** |  | **.552**** |
| Verbal Fluency | **.272*** | -0.234 | **.389^**^** | **.262^*^** | **.314^*^** | **.565^**^** |  |

Supplementary Table 1: Correlations Between Measures
